# Implications of Post-Translational Modifications in Autoimmunity with Emphasis on Citrullination, Homocitrullination and Acetylation for the Pathogenesis, Diagnosis and Prognosis of Rheumatoid Arthritis

**DOI:** 10.3390/ijms232415803

**Published:** 2022-12-13

**Authors:** Isabel Haro, Raimon Sanmartí, María J. Gómara

**Affiliations:** 1 Unit of Synthesis and Biomedical Applications of Peptides, Institute for Advanced Chemistry of Catalonia Consejo Superior de Investigaciones Científicas (IQAC-CSIC), Jordi Girona 18-26, 08034 Barcelona, Spain; 2 Arthritis Unit, Rheumatology Department, Hospital Clinic of Barcelona, Villarroel 170, 08036 Barcelona, Spain

**Keywords:** post-translational modification, citrullination, homocitrullination, acetylation, ACPA, AMPA, rheumatoid arthritis, synthetic peptides

## Abstract

Post-translational modifications (PTMs) influence cellular processes and consequently, their dysregulation is related to the etiologies of numerous diseases. It is widely known that a variety of autoimmune responses in human diseases depend on PTMs of self-proteins. In this review we summarize the latest findings about the role of PTMs in the generation of autoimmunity and, specifically, we address the most relevant PTMs in rheumatic diseases that occur in synovial tissue. Citrullination, homocitrullination (carbamylation) and acetylation are responsible for the generation of Anti-Modified Protein/Peptide Antibodies (AMPAs family), autoantibodies which have been implicated in the etiopathogenesis, diagnosis and prognosis of rheumatoid arthritis (RA). Synthetic peptides provide complete control over the exact epitopes presented as well as the specific positions in their sequence where post-translationally modified amino acids are located and are key to advancing the detection of serological RA biomarkers that could be useful to stratify RA patients in order to pursue a personalized rheumatology. In this review we specifically address the latest findings regarding synthetic peptides post-translationally modified for the specific detection of autoantibodies in RA patients.

## 1. Post-Translational Modifications in Proteins

Post-translational modifications (PTMs) are reversible or irreversible chemical reactions that occur in specific amino acids (aas) of some proteins after their translation. In fact, more than 140 different aa-based structures can constitute the proteins when considering the PTMs that could modify the 20 natural aas [1]. Several factors, such as the location of the aa within the protein’s primary sequence, will determine whether PTMs occur as well as their type and frequency.

PTMs are not encoded by the cellular genome and can significantly modify protein structure and modulate functions such as folding, degradation, signaling, localization, stability, enzymatic activity and protein-protein interactions [2].

**Figure 1:**
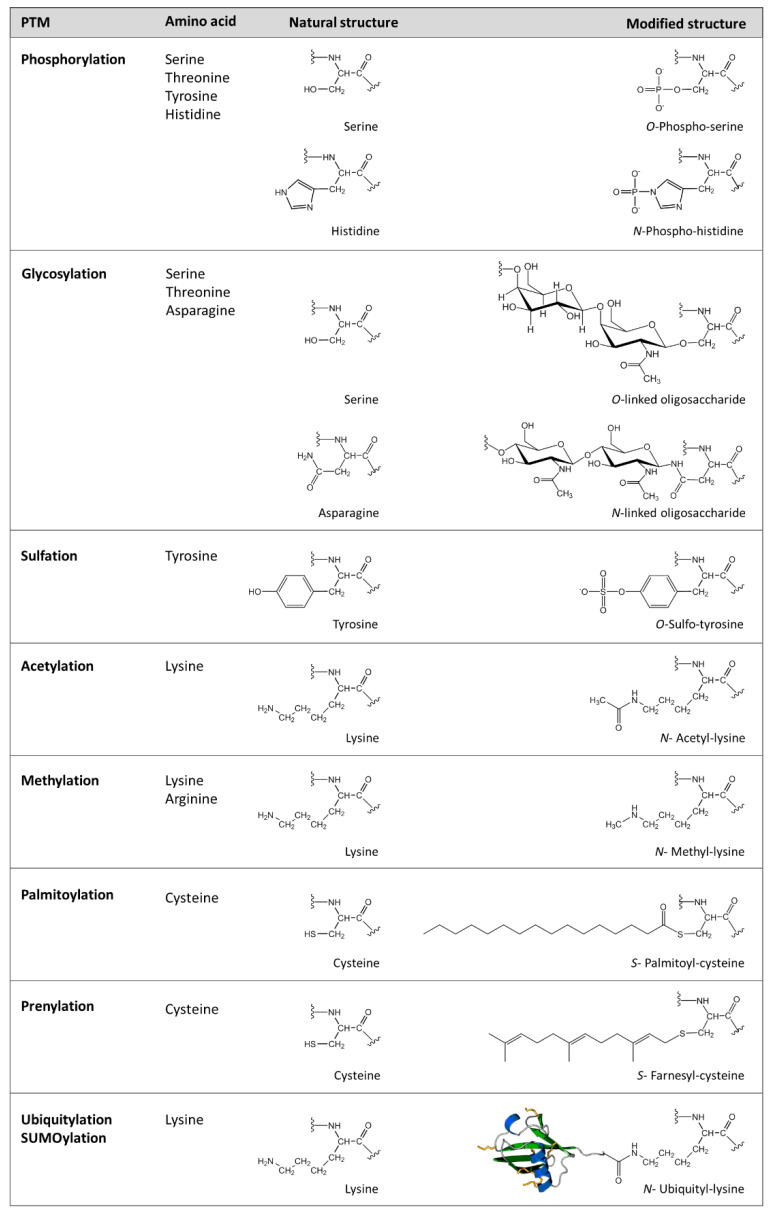
Natural and modified structures of amino acids that undergo the most frequent PTMs. His phosphorylation is understudied compared to that of Ser, Thr or Tyr.

More than 400 different types of PTMs have been described in humans occurring in several cellular organelles (nucleus, cytoplasm, endoplasmic reticulum and Golgi apparatus). They are involved in a large number of cellular events and are considered a key mechanism for the regulation of the biological activity of proteins. There are 24 most frequent PTMs, phosphorylation, acetylation and ubiquitination being the ones that occur most frequently have been better studied (Figure 1). Accordingly, multiple modifications at specific residues converge, Lysine being the aa undergoing most PTMs (15 PTMs) [3].

PTMs have been studied and detected since many years ago using classic approaches which mainly involved Edman degradation and thin-layer chromatography; however, the use of mass spectrometry has changed the paradigm and enormously contributed to identifying thousands of novel PTM sites. Thus, PTMs are nowadays routinely identified and quantified by mass spectrometry because most of them result in a change in the mass of the modified protein. Farley and Link discuss in an excellent revision the existing and emerging techniques in the field of mass spectrometry and proteomics that are available to identify and quantify PTMs [4]. Of note, publicly accessible online databases of PTMs exist, dbPTM (Database Post-translational modification) being the largest one, not only in the number of recorded proteins but also in the stored PTM types [3]. The main PTM databases as well as the computational methods to predict PTMs that have been developed in recent years are comprehensively reviewed in [5,6].

As described, PTMs influence cellular processes and consequently, their dysregulation is related to the etiopathogenesis of numerous diseases [2]. Neurodegenerative diseases and cancer are the principal diseases affected by dysregulation of PTMs [7,8] and ubiquitylation, prenylation, glycosylation, S-palmitoylation and SUMOylation are the PTMs that are the most present in diseases [5]. Furthermore, a variety of autoimmune responses in human diseases depend on PTMs of self-proteins. Table 1 shows some of the most relevant target proteins that undergo PTM in dysregulation processes that are associated with human immune-mediated diseases.

## 2. PTMs in the Generation of Autoimmunity

In the early 2000s, Doyle and Mamula clearly addressed the question of how PTMs of proteins affect the processing of foreign and self-antigens and what their role is in the origin of autoimmune responses [26]. They shed light with explanatory examples on the role of PTMs in autoimmunity and detailed how protein modifications may alter protein biology, processing and presentation of autoantigens by the immune system as well as the mechanisms of the breakdown of tolerance by post-translationally modified proteins [27].

In the context of disease and inflammation, the generation of PTMs in human proteins is usually the consequence of a physiological response to cellular stress that can cause the release of cellular products such as reactive oxygen species (ROS) and enzymes that promote the modification of amino acid residues. The production of ROS at physiological levels contributes to the resolution of inflammation and the maintenance of homeostasis in tissues. However, when ROS release is excessive, these levels can increase to the point of overcoming the antioxidant capacity of natural defenses that are unable to eliminate excess ROS. In such conditions of oxidative stress, apoptosis processes also have a role in the generation of self-antigens since self-proteins are cleaved differently in apoptotic cells. Furthermore, these proteins are subject to several modifications such as phosphorylation, transglutamination, ubiquitination and citrullination. Therefore, cells undergoing apoptosis are considered rich sources of PTM-altered self-proteins [28]. 

The modified proteins can trigger the formation of new epitopes (neoepitopes) that are able to induce robust autoimmune responses altering the immune tolerance to self-proteins. The neoepitopes are different from the epitopes of self-proteins to which the immune system is already tolerant, becoming “foreign” to the organism and breaking tolerance of the immune system. In addition, it has been described that autoimmune responses originating from the post-translationally modified self-proteins diversify in an intra- and extra-molecular manner to other self-protein epitopes [14]. Thus, breaking immune tolerance to a PTM self-protein promotes “epitope spreading”, a mechanism where autoimmune response may be amplified involving other epitopes beyond the one which initiated the response. PTMs in self-proteins can profoundly affect the recognition of antigens by the antigen-presenting cells with the subsequent alteration of the specificity of B- and T-lymphocyte immunity. As described by Valesini et al. [29], small modifications in the structure can alter the immunogenicity of the proteins due to an enhanced protein unfolding and subsequent processing and exposure of the immunogenic epitopes [30]. 

As pointed out by Van Damme’s research group, autoimmunity research has been classically centered on the study of antigen presentation to T lymphocytes, the role of B lymphocytes and the antibody recognition of autoantigens. In addition to the adaptive immunity, these authors highlight the importance of the innate immune system in the generation of autoimmunity and propose that extracellular proteolysis, regulated by cytokines and proteases from innate immune cells, generates autoantigens that start autoimmune reactions [31]. Cytokine-regulated extracellular proteolysis and other PTMs of autoantigens can be executed by host enzymes, including complement molecules, matrix metalloproteinases and deiminases, demonstrating the prominent role that these molecules and innate immune cells play in autoimmune diseases [32]. 

In addition to the immune system, host genetics and environmental factors play also major roles in autoimmune pathogenesis and progression. 

Regarding genetics, heritable genetic risk traits have been defined by Genome-Wide Association Studies (GWAS) in many autoimmune diseases. Recently, Caliskan et al. have reported a comprehensive catalog of GWAS fine mapping in autoimmune diseases with the aim of predicting the probability of mediating disease risk associations across genes in GWAS loci and identifying robust signals of causal disease biology [33]. 

Among environmental factors, microbes (bacteria and viruses) can induce PTMs that can contribute to autoantigenic neoepitope formation. Microorganisms can directly provide proteases or other enzymes that can modify specific amino acids in proteins or peptides via glycosylation or citrullination among other changes, which can contribute to the generation of autoantigens stimulating T cells. [34,35,36] One of the most-reported links between bacterial infection and autoimmune diseases through PTM is related to *Porphyromonas gingivalis*, the major pathogen of periodontal disease, with an increased prevalence in rheumatoid arthritis (RA) [37,38]. This prokaryote expresses the bacterial peptidylarginine deiminase (PAD) which generates citrullinated epitopes distinct from those generated by endogenous PADs, thus contributing to aggravation of RA [35]. More recently, in the context of the SARS-CoV-2 pandemic, it has been speculated that coronavirus infection might exacerbate the formation of PTMs and, in so doing, provoke the onset of type 1 diabetes mellitus [36]. Furthermore, Epstein-Barr virus (EBV) infection has been epidemiologically linked to multiple sclerosis [39]. High-affinity molecular mimicry between EBV nuclear antigen 1 (EBNA1) and glial cell adhesion molecule (GlialCAM) has been recently demonstrated, which is facilitated by post-translational phosphorylation of the central nervous system protein GlialCAM [40]. These latest findings highlight the role of viruses in autoimmune diseases and deserve further investigation to unravel the involvement of viruses in the processes that lead to inflammation.

## 3. PTMs in Inflammatory Immune-Mediated Rheumatic Diseases

Inflammatory rheumatic diseases are autoimmune and/or immune-mediated diseases caused by the immune system’s own attack on the joints, muscles, bones and organs. They include a variety of disorders involving synovial joints; among them RA and spondyloarthropathies (including psoriatic arthritis) are the most common, accounting for a large percentage of disability [41]. RA affects 0.5–1% of adults worldwide, with women being three times more susceptible than men. The chronic polyarthritis causes joint destruction and deformities, together with functional disability and reduced quality of life and life expectancy [42]. In RA there is a persistent activation of the immune system accompanied by inflammation, especially in the joints (synovitis), one of the main characteristics of said inflammation being ROS. This imbalance towards an excess of oxidant molecules (oxidative stress) is closely related to RA. Moreover, an interesting and enigmatic entity with a close, but not well understood, relationship with RA is Palindromic Rheumatism (PR), a form of relapsing/remitting arthritis that may evolve into RA. Although its frequency is significantly lower than that of RA it is clearly considered a pre-RA stage for most patients. PR autoimmunity plays a substantial role in this rheumatic disease, with the same characteristic autoantibody profile observed in RA, although with some differences in the immune response repertoire [12]. 

**Figure 2:**
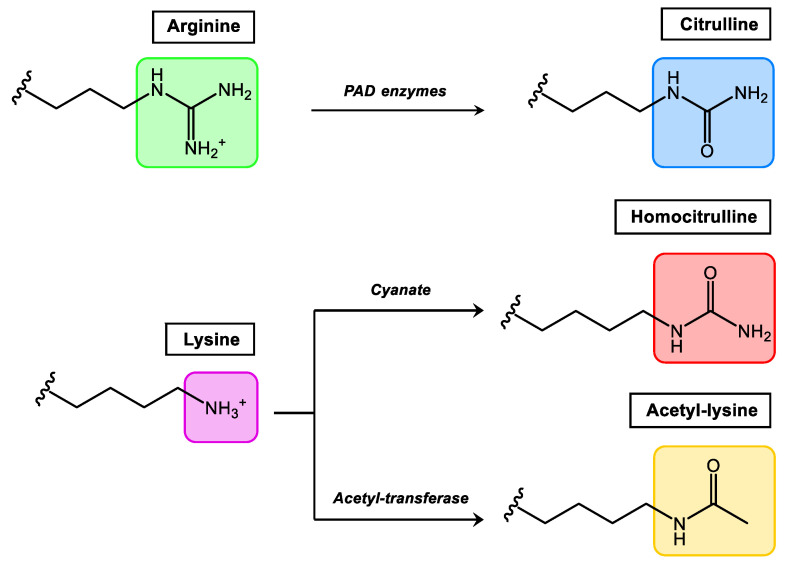
Relevant PTMs in inflammatory rheumatic diseases.

Comprehensively studied PTMs that occur in synovial tissue and at present considered more relevant in rheumatic diseases are citrullination, homocitrullination (carbamylation) and acetylation (Figure 2). Among them, citrullination is the best-studied PTM in rheumatology, and Anti-Citrullinated Protein/Peptide Antibodies (ACPAs) were included by the European League against Rheumatism (EULAR)/American College of Rheumatology (ACR) as classification criteria for RA in 2010 [43].

Citrullination, which consists of the conversion of arginine to the non-essential amino acid citrulline, is mediated by the enzyme peptidylarginine deiminase which is found in the inflamed synovium. Homocitrullination or carbamylation is a non-enzymatic PTM that involves the conversion of lysine residues to homocitrullinated residues after a reaction with cyanate. Acetylation is a reversible enzymatic process (balance between acetylases and deacetylases) where acetyl groups are added to free amines of lysine residues. In general terms, all autoantibodies found in RA belong to the Anti-Modified Protein/Peptide Antibodies (AMPAs) family.

Other autoantigens including malondialdehyde (MDA) and malondialdehyde-acetaldehyde (MAA) adduct containing proteins [44] or autoantibodies targeting the other four PTMs, such as chlorination, non-enzymatic glycation, nitration and homocysteinylation [45] have been recently studied in RA. Specifically, an increased lipid peroxidation is produced when tissues are exposed to oxidative stress, and this results in the formation of MDA which degrades into acetaldehyde and both are also able to react and form the more stable protein adduct MAA. However, despite the initially surprising described high sensitivity of anti-MAA antibodies for RA [44], these autoantibodies are not specific for RA, having been implicated in other diseases associated with increased oxidative stress and lipid peroxidation and detected in patients with other rheumatic conditions [46], cardiovascular diseases [47], liver damage [48], type II diabetes mellitus [49] or smoking-related diseases [50]. Recently, Rodriguez-Martinez et al. [45] reported only significant differences of anti-glycated collagen type II autoantibodies in RA patients, although the magnitude of the specific signal was very small, which led the authors to conclude that the four atypical antigens included in their analysis were not able to induce significant autoantibody response in RA patients, thus indicating that the repertoire of PTM autoantigens in RA is restricted.

Furthermore, N-glycosilation or O-glycosilation, the addition of glucides on an atom of nitrogen or oxygen of the lateral chain of the amino acid proteins, are mediated by hundreds of glycosyl-transferases and aberrant glycosylation of IgG has been implicated in RA pathogenesis [51,52]. 

ACPA target proteins include mainly endogenous autoantigens that are expressed in organs and tissues implicated in the immunopathology of RA [53,54,55]. Apart from filaggrin, one of the first citrullinated proteins identified as an ACPA target that is expressed in the epithelium [56], other highly expressed and citrullinated proteins have been described in inflamed synovium. These citrullinated antigens include structural components of the joints, such as type II collagen, α-enolase and fibronectin, proteins that form deposits in inflamed joints, mainly fibrinogen/fibrin and vimentin [57,58,59], as well as other antigens that have been identified in synovial fluid from RA patients (apolipoprotein E, β-actin and myeloid nuclear differentiation antigen or MNDA) [60]. In addition, intracellular proteins (immunoglobulin-binding protein, BIP) [61] or intranuclear proteins, [62,63,64] such as histones, chaperones and heterogeneous nuclear ribonucleoproteins (hnRNP), have also been described as targets of ACPAs in inflammatory conditions. These nuclear proteins become accessible to the immune system in the Neutrophil Extracellular Traps (NETs) [65] after being extruded by NETosis processes, which are another form of cell death different from apoptosis and necrosis. 

## 4. AMPAs in the Pathogenesis, Diagnosis and Prognosis of RA

The pathogenesis of RA has not yet been fully defined, with genetic, environmental and immunological factors being involved in the development of RA [66].

The study of the genetic factors predisposing for RA was suggested more than 30 years ago; the association between RA and HLA-DRB1*04 being first reported by Stastny [67]. In this complex multigenic pathology, genetic factors may account for as much as 60% of disease susceptibility [68]. The most important genetic factor is Shared Epitope (SE) of Human Leukocyte Antigen (HLA-DRB1) of the Major Histocompatibility Complex (MHC) [69]. This SE motif (QKRAA, QRRAA or RRRAA) is encoded by HLA-DRB1*04 (*0401, *0404, *0405 and *0408 alleles) and some non-DR4 alleles, such as HLA-DRB1*0101, HLA-DRB1*1402 and HLA-DRB1*1001. HLA-DRB1*0401 is related to the strongest association with disease susceptibility and severity [70]. However, other genetic factors exist in non-HLA regions (recently reviewed in [66]) and also different epigenetic mechanisms such as DNA methylation changes are important in RA pathogenesis [71]. 

Environmental factors (smoking, air pollutants, obesity and infections) are able to trigger RA in individuals who are genetically predisposed. 

Cigarette smoking is the main environmental factor associated with PTMs in proteins and it has been reported as a clear risk factor for breaking tolerance to multiple autoantigens in RA [72]. Although it was related to the production of citrullinated autoantibodies, the inflammation and damage of lung mucosa and the development of a more severe RA disease [73], it seems that it is not only associated with the presence of ACPA, but rather with the concurrent presence of several autoantibodies in RA, since citrullination is not the only PTM that can be enhanced by tobacco. Smoking or the presence of pathogenic bacteria directly implicated in periodontitis cause lung and oral inflammation and the generation of PTMs and autoantigens that in susceptible subjects are able to initiate the autoimmune phase of RA. Moreover, an increase in pathogenic bacteria and a decrease in the beneficial ones (mucosal dysbiosis) have been detected in RA patients, although the data about its involvement in the pathogenesis of RA are inconclusive. Holers et al. published an excellent review on the mucosal origin hypothesis for RA, which would indicate that RA development could begin at mucosal sites and then proceeds to involve synovial joints [74]. 

The pathogenic potential of autoantibodies in RA is also a matter of debate. The presence and expansion of these autoantibodies in preclinical phases before disease onset as well as its association with joint destruction strongly suggest a causative role in the disease’s development and progression. However, the presence of autoantibodies in synovial tissues alone is not enough to cause synovitis. An additional stimulus (i.e., immune complex formation or complement activation) is probably required to initiate clinical RA. We refer the readers to recent excellent reviews about the contribution of autoantibodies to etiopathogenesis of RA [75,76,77]. 

To prevent progressive and irreversible structural damage, the identification of individuals at high risk of developing RA is of paramount importance. However, diagnosis is difficult in the early stages of RA, because symptoms are not always present and there is no single discriminatory test to clearly diagnose and/or classify most rheumatic diseases. In the clinical context, ACPAs are determined by using commercial tests and their presence in established disease has been associated with a worse prognosis. They can be present several years before disease onset, which occurs with an increase in ACPA levels and a further epitope spreading. Once reaching sufficient levels for an RA diagnosis, ACPAs remain stable over time with small changes in levels and only occasionally do patients become negative on the test [78].

## 5. Synthetic Post-Translationally Modified Peptides for the Specific Detection of Autoantibodies in RA Patients

ACPAs can be detected using immunoassays with different citrullinated proteins or peptides as antigenic substrates. As a summary, Table 2 illustrates the most common citrullinated peptide antigens that have been used in RA diagnosis. 

**Figure 3:**
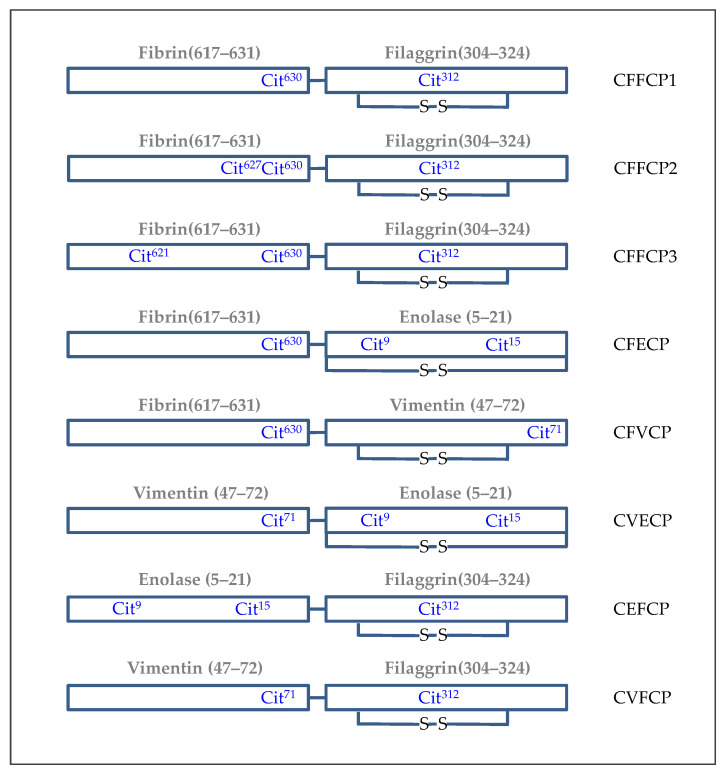
Scheme of cyclic chimeric citrullinated peptides derived from different proteins present in the rheumatoid synovial fluid. The protein domains and the citrullination positions are indicated for each cyclic chimeric peptide.

Unlike citrullinated proteins, synthetic citrullinated peptides provide complete control over the exact epitopes presented and the specific positions in their sequence where post-translationally modified amino acids are located. They are unambiguously defined chemical molecules that mimic segments of complex protein antigens involved in autoantibody binding and are easily obtained in a pure form [79]. Based on these advantages, Shellekens et al. described synthetic citrullinated peptides as antigens for the specific detection of ACPAs in RA patients [80] and developed in 2000 the first-generation ELISA test (CCP1) based on a filaggrin-derived cyclic citrullinated peptide [81]. Since then, second- and third-generation tests (CCP2 and CCP3/CCP3.1) have been launched on the market and widely used in clinical practice. These commercial tests are based on cyclic peptides with unpublished primary structures. Our research group reported the epitope mapping of α- and β-fibrin and vimentin proteins working with libraries of citrullinated synthetic peptides that were obtained following Solid Phase Peptide Synthesis (SPPS) strategies [82,83,84]. We were able to select several reactive domains of these two proteins and subsequently, the peptides were covalently bound to the previously published enolase and filaggrin peptides [85,86] to render the Citrullinated Fibrin-Filaggrin Chimeric Peptides (CFFCPs) that are shown in Figure 3. The presence of different peptide sequences within the same molecule resulted in synergistic effects compared to the monomeric peptides or the corresponding physical mixtures of them. Thus, the chimeric peptides allowed a detailed analysis of the autoantibodies’ multi-reactivity found in the sera of patients suffering from this heterogeneous disease. Autoantibodies against CFFCP1, CFFCP2 and CFFCP3 showed a comparable sensitivity and specificity for RA to CCP2 and CCP3/CCP3.1 commercial tests and rendered better results in terms of identifying patients with poor radiographic outcomes [87,88].

The features of broad heterogeneity of ACPAs were reported by illustrating different reactivity patterns of RA patients’ sera against a high number of citrullinated proteins and peptides in different studies. Hence, a large number of citrullinated peptides were identified as ACPA targets using multiplex technology, the work of Hueber et al. [89] being one of the first that described multiplex ACPA profiling assays using synovial proteome microarrays containing 225 peptides and proteins. More recently, the works of Wagner et al. [90] and Ronnelid et al. [91] developed multiplex assays to identify RA patients with distinct genetic and environmental determinants that were missed by the commercial test. Garcia-Moreno et al. [92] reported 100% specificity for the detection of ACPAs in a citrullinated chimeric-peptide-based multiplex platform (Figure 3) in which the array positivity was defined as being positive for more than three peptides and the control groups consisted of blood donors and patients affected by psoriatic arthritis, an inflammatory disease whose clinical presentation can simulate that of RA. Multiplexing techniques demonstrated that the simultaneous analysis of target citrullinated peptides facilitates the identification of patient subgroups with special clinical characteristics.

**Table 2:** Citrullinated peptides as targets of ACPAs.

Protein	Sequence	PTM Peptide	References
Filaggrin	(306–324) (304–324)	SHQEST**Cit**GRSRGRSGRSGS (cyclic) HQCEST**Cit**GRSRGRCGRSGS (cyclic) HQCHQEST**Cit**GRSRGRCGRSGS (cyclic) HSGIGHGQASSAVRDSGH**Cit**GYS HSTSQEGQDTIHGH**Cit**GS	[81,93] [94,95,96,97,98] [81,91,99] [55] [55]
Profilaggrin	(293–310)	TIHAHPGS**CitCit**GGRHGYHH TIHAHPGS**Cit**RGG**Cit**HGYHH	[100]
Fibrinogen (α-chain)	(27–43) (31–50) (36–50) (41–60) (84–96) (211–230) (556–575) (563–583) (580–600) (616–636) (617–631) (621–635)	FLAEGGGV**Cit**GPRVVERH GGGV**Cit**GPRVVERHQSACKDS GP**Cit**VVE**Cit**HQSASKDS E**Cit**HQSACKDSDWPFCSDEDW (cyclic) TSSTSYN**Cit**GDSTF DLLPS**Cit**DRQHLPLIKMKPVP (cyclic) NTKESSSHHPGIAEFPS**Cit**GK (cyclic) HHPGIAEFPS**Cit**GKSSSYSKQF SKQFTSSTSYN**Cit**GDSTFESKS THSTK**Cit**GHAKS**Cit**PV**Cit**DCDDVL (cyclic) HSTKRGHAKSRPV**Cit**G **Cit**GHAKS**Cit**PV**Cit**GIHTS	[56,95] [100] [86,91,93,94,98,101] [102] [95] [102] [97,102] [91,93,94,96,98,103] [91,93,94,98,103] [100,102] [83] [86,91,93,94,96,98,101]
Fibrinogen (β-chain)	(36–52) (43–62) (60–74) (54–80) (62–81) (62–81)	NEEGFFSA**Cit**GHRPLDKK ARGH**Cit**PLDKKREEAPSL**Cit**PA **Cit**PAPPPISGGGY**Cit**A**Cit** EEAPSL**Cit**PAPPPISGGGY**Cit**A**Cit**PAKAAA APPPISGGGY**Cit**ARPAKAAAT APPPISGGGYRA**Cit**PAKAAAT	[56,91,93,94,96,97,98] [83] [86,91,93,94,96,98,101] [95] [94,96,97,103] [94,96,97,103]
Fibronectin	(1029–1042) (2350–2362)	LTVGLT**CitCit**GQPRQY YNQYSQ**Cit**YHQRTN	[95] [95]
Vimentin	(2–17) (47–72) (59–70) (60–75) (265–278) (420–435)	ST**Cit**SVSSSSY**CitCit**MFGG STSRSLYASSPGGVYATRSSAVRL**Cit**S GRVYAT**Cit**SSAVR VYATRSSAVRL**Cit**SSVP LTAAL**Cit**DV**Cit**QQYES SLNL**Cit**ETNLDSLPLVD	[56,93,94,96,97,98] [84] [104,105] [56,93,94,96,97,98] [95] [95]
α-enolase	(5–21)	CKIHA**Cit**EIFDS**Cit**GNPTVEC (cyclic)	[56,85,91,93,94,95,96,97,98]
Collagen type II Collagen type II (citC1^III^)	(282–298) (359–369)	GLPGVKGH**Cit**GYPGLDGA (GPO)_5_-GA**Cit**GLTG**Cit**PGDA(GPO)_2_-GKKYG	[95] [91,96,97,98]
Biglycan	(247–266)	EDLL**Cit**YSKLY**Cit**LGLGHNQIR (cyclic)	[90,102]
Clusterin	(221–240) (334–353)	PKS**Cit**IV**Cit**SLMPFSPYEPLNF (cyclic) AE**Cit**LT**Cit**KYNELLKSYQWKML	[90,102] [100]
β-actin	(191–216)	MKILTE**Cit**GYSFTTAE**Cit**EIV**Cit**DIKEKL	[95]
MNDA	(121–135)	KLTSEA**Cit**G**Cit**IPVAQK	[95]
Histone 2A Histone 2B	(1–20) (62–81)	MSG**Cit**GKTGGKA**Cit**AKAKS**Cit**SS (cyclic) IMNSFVNDIFE**Cit**IAGEAS**Cit**L (cyclic)	[90,102] [90,102]
Histone 4	(33–47)	AIRRLA**CitCit**GGVLRIS	[62]
Tenascin C	(2176–2200)	EHSIQFAEMKL**Cit**PSNF**Cit**NLEG**CitCit**KR	[106]

Collagen type II (citC1^III^): triple-helical type II collagen peptide C1; GPO: glycine-proline-hydroxyproline repeats; MNDA: myeloid nuclear differentiation antigen.

Recently, it has been described that antibodies recognizing citrullinated proteins display a broad cross-reactivity towards other PTMs [93,94,99]. Sahlström et al. used three novel peptide-screening platforms with RA autoantigens, citrullinated and carbamylated peptides, or histone derived peptides with different PTMs (covering >207,000 peptides) to characterize the multi-reactivity of RA-patient-derived monoclonal ACPAs [94]. The observed monoclonal ACPAs’ multi-reactivity to a large number of citrullinated peptides/proteins was explained by the recognition of certain consensus peptide motifs that are present across multiple proteins. In addition, for some ACPA clones, this multi-reactivity was also extended to PTMs of lysine residues, specifically carbamylation and acetylation. As demonstrated by Kissel et al. [99], B cells directed against a particular PTM can be activated by other PTM antigens in inflamed tissues or other sites involved in the breach of B-cell tolerance. Thus, autoantibodies recognizing other post-translationally modified antigens, such as anti-carbamylated protein antibodies (anti-CarP) and anti-acetylated protein antibodies (anti-APA), have also been identified in RA patients [11,104,107]. In general terms, all autoantibodies found in RA show a broad reactivity to various antigenic backbones and are highly cross-reactive towards at least two different PTMs [99]. Table 3 shows peptide sequences with PTMs different from citrullination that have shown reactivity with autoantibodies in RA patients. 

Grönwall et al. analyzed the relationship between IgG/IgA AMPAs’ specificities and citrulline-, homocitrulline-, acetyl-lysine- and MAA-modified antigens in a panel of nearly 2000 RA patients. The authors found auto-reactivity to carbamylated and acetylated epitopes in RA patients with citrulline reactivity and observed reactivity to acetyl-lysine in a substantially smaller patient subset mostly overlapping with citrulline and homocitrulline reactivity. Otherwise, the anti-MDA/MAA reactivity was not RA-specific and showed low correlation with ACPA fine specificities. ACPAs and autoantibodies that recognize homocitrulline and acetyl-lysine represent overlapping facets of RA autoimmunity [93,94]. 

**Figure 4:**
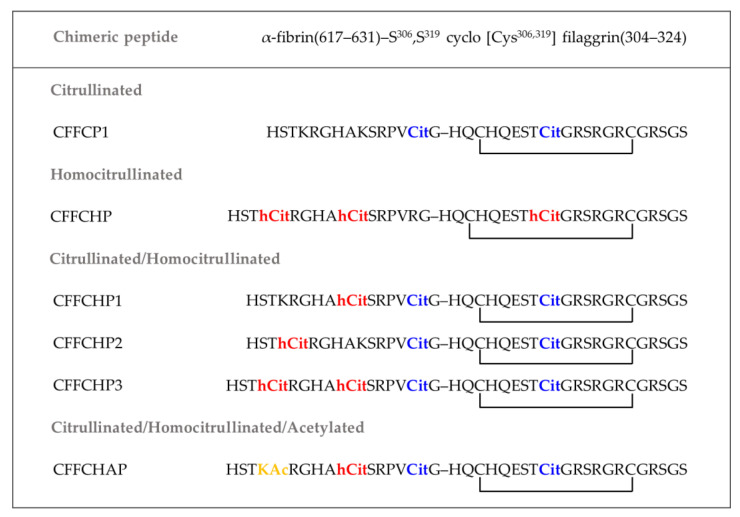
Primary structure of cyclic chimeric peptides bearing one, two and three PTMs in their sequence. Cit: citrulline; hCit: homocitrulline; KAc: acetyl-lysine.

Anti-CarP are present in about 45% of RA patients as well as in 10–20% of patients previously considered ACPA-negative [107] and have also been found in patients with palindromic rheumatism (PR) but in a lower proportion and with a different isotype usage than in RA, suggesting a distinct B-cell response to homocitrullinated antigens in PR [113]. Anti-CarP have been associated with poor disease outcomes in RA patients [107], including higher disease activity and radiographic progression, and have been detected in various chronic lung diseases [114]. Castellanos et al. [115] described for the first time in the literature that anti-CarP that recognize homocitrullinated peptides and proteins are strongly associated with interstitial lung disease (ILD), suggesting a possible link between homocitrullination and the development of ILD in RA patients. A synthetic Chimeric Fibrin/Filaggrin Homocitrullinated Peptide (CFFCHP, Figure 4) as well as Fetal Calf Serum (FCS) and Fibrinogen carbamylated proteins were used as antigens to determine the prevalence and isotype usage of anti-CarP in ILD-RA patients. The association between ILD and anti-CarP presented the highest odds ratio for the IgA isotype, supporting the hypothesis that RA-related autoantibodies might originate in the respiratory mucosa. These findings can contribute to the early detection of ILD, an extra-articular manifestation that entails a high mortality, which is crucial in establishing an appropriate treatment strategy. 

Considering that the overlap between the three families of autoantibodies (ACPAs, anti-CarP and anti-APA) is characteristic of RA, novel peptide-based antigens bearing citrulline, homocitrulline and/or acetyl-lysine were designed to further study the role of AMPAs as biomarkers linked to the presence of ILD as well as the severity of joint destruction [116]. Based on the previously defined synthetic peptide structures (CFFCP1 and CFFHP), three novel citrullinated/homocitrullinated (CFFCHP1, CFFCHP2 and CFFCHP3) and one citrullinated/homocitrullinated/acetylated (CFFCHAP) chimeric peptides were synthesized by SPPS preserving the citrulline at the 630 position of the α-fibrin (617–631) domain, taking into account the important role of this specific position for autoantibody binding that was previously reported [83,86]. The synthetic antigens were used to compare the reactivity of the three different PTMs in RA patients using the same peptide backbone. In contrast to PTM-protein antigens that usually lead to heterogeneous antigen epitopes, this strategy allows us to consistently test the AMPAs’ reactivity against a chemically well-defined antigen. The association of the different AMPAs with a severe clinical phenotype of RA was confirmed with the different autoantibodies, and especially for the IgA and IgM isotypes. Regarding the IgA isotype, the presence of two or three PTMs was able to detect a percentage close to 20% of RA-ILD sera that were negative when analyzed with the peptide bearing a single PTM. In the same line, Niijar et al. [105] recently reported that the optimal prediction of erosive radiographic progression in patients with new-onset RA requires study of the effect of AMPA combinations.

To the best of our knowledge, in Ref. [116] is reported for the first time in the literature a peptide that simultaneously contains the three most relevant PTMs for RA at specific positions of their unique primary structure as an antigenic target of the autoantibodies associated with severe clinical phenotypes of RA. 

## 6. Concluding Remarks

The presence of RA-specific PTMs such as citrulline, homocitrulline and acetyl-lysine on the same antigenic peptide backbone may provide valuable information on multiple-autoantibody specificities in RA patients. Scarce data are available on the importance of AMPA combinations and given the heterogeneity of the autoantibody response in RA patients, further studies with peptide antigens bearing multiple PTMs could provide important insights to relate AMPAs with the patients’ immune activity, early RA diagnosis and RA progression. A detailed analysis of AMPA profiles would allow establishment of associations with RA severe clinical phenotypes in order to improve the diagnosis and the treatment of serious RA complications. In addition, the study of the different AMPA fine specificities as biomarkers of therapeutic response to anti-rheumatic therapies may have implications in future clinical practice. Further work to understand the effect of multi-reactivity in RA patients is warranted. 

The use of well-defined peptide antigens that present multiple PTMs is key to advancing the detection of serological biomarkers that could be useful to stratify RA patients in order to pursue a personalized rheumatology.

## Figures and Tables

**Table 1:** General overview of PTMs in the main target proteins related to human immune-mediated diseases.

Human Disease	Target Proteins	PTM	References
Multiple Sclerosis	Myelin proteins (MBP), GFAP, Histone H3	Citrullination	[9]
Rheumatoid Arthritis Palindromic Rheumatism	Proteins of inflamed joints and synovium: Collagen type II, Fibrinogen, Fibrin, Vimentin, α-Enolase	Citrullination Carbamylation Acetylation Glycosylation	[10,11,12,13]
Type 1 Diabetes	Proteins modified in beta-cells and neutrophils (IAPP, IA-2, IGRP, GAD65, GRP78)	Citrullination	[14]
Systemic Lupus Erythematosus	Intracellular macromolecules: ribonucleoproteins and nucleosomes (U1-70K, Ro/SSA, La/SSB, SmD1, H2B, H4, …)	Phosphorylation Glycosylation Acetylation Iso-aspartylation Methylation Ubiquitination	[15,16,17]
Celiac disease	Adherence and tight junction proteins; transcription factor (PPARγ); serum IgG and IgA1 Wheat gliadin	Phosphorylation Ubiquitination Glycosylation Citrullination	[18,19]
Psoriasis/Psoriatic arthritis	Keratin; Cathelicidin LL37	Citrullination Carbamylation	[20,21]
Antiphospholipid Syndrome	Apolipoprotein (β2GPI), Vimentin	Oxidation/ Sialylation Citrullination	[22]
Primary Biliary Cirrhosis	PDC-E2	Acylation/ oxidation	[23]
Inflammatory Bowel Disease	Kinases: MAPKs and PKB/Akt; DNA-binding transcription factor; NF-κB; Histone H3	Phosphorylation Ubiquitination Glycosylation Citrullination	[19,24]
Sjogren’s Syndrome	α-Enolase	Citrullination	[25]

Myelin basic protein (MBP); glial fibrillary acidic protein (GFAP); Islet amyloid polypeptide (IAPP); Islet antigen-2 (IA-2); Islet-specific glucose-6-phosphatase catalytic (IGRP); glutamic acid decarboxylase (GAD65); glucose-regulated protein 78 (GRP78); Histone H2B; Histone H4; Peroxisome proliferator-activated receptor gamma (PPARγ); β2-glycoprotein I (β2GPI); E2 subunit of the pyruvate dehydrogenase complex (PDC-E2); Mitogen-activated protein kinases (MAPKs); Nuclear factor-kappa B (NF-κB); Signal transducer and activator of transcription 3 (STAT3); Protein kinase B (PKB/Akt).

**Table 3:** Post-translationally modified peptides as targets of AMPAs: PTM-peptide sequences from different proteins that have shown cross-reactivity with AMPAs in RA patients.

Protein	PTM	Peptide Sequence	References
Filaggrin	Homocitrulline Acetyl-Lysine	HQCHQEST**hCit**GRSRGRCGRSGS HQCHQEST**KAc**GRSRGRCGRSGS	[99]
Vimentin	Homocitrulline Acetyl-Lysine Acetyl-ornithine	GRVYAT**hCit**SSAVR GRVYAT**KAc**SSAVR GRVYAT**OrAc**SSAVR	[93,104,105,108,109]
Vimentin	Homocitrulline Acetyl-Lysine	VYAT**hCit**SSAV**hCit**L**hCit**SSVP VYAT**KAc**SSAV**KAc**L**KAc**SSVP	[99]
Fibrinogen α	Homocitrulline Acetyl-Lysine	FLAEGGGV**hCit**GPRVVERH FLAEGGGV**KAc**GPRVVERH	[99]
Fibrinogen β	Homocitrulline	ARGHRPLDK**hCit**REEA	[93,109]
Fibrinogen β	Homocitrulline	AKAAATQ**hCit**KVER (cyclic)	[93,109]
Fibrinogen β	Homocitrulline Acetyl-Lysine	NEEGFFSA**hCit**GHRPLDKK NEEGFFSA**KAc**GHRPLDKK	[99]
Fibrinogen *γ*	Carbamylation	QKIVNLKEKVAQLEAQCQEPCKDTVQI * WMNKCHAGHLNGVYYQGGTYSKASTPN *	[110]
Enolase	Homocitrulline Homocitrulline Acetyl-Lysine	KIHA**hCit**EIFDS**hCit**GNPTVE (cyclic) KIHA**hCit**EIFDS**hCit**GNPTV (linear) KIHA**KAc**EIFDS**KAc**GNPTV (linear)	[93,111] [99]
Histone 2B	Acetyl-Lysine	SAPAPK**KAc**GSKKAVTKAQ (cyclic)	[93,112]
Histone 4	Acetyl-Lysine Acetyl-Lysine Acetyl-Lysine	SGRG**KAc**GG**KAc**GLG**KAc**GGA**KAc**RH SGRG**KAc**GGKGLGKGGAKRH SGRGKGGKGLGKGGA**KAc**RH	[93,112]

hCit: homocitrulline; KAc: acetyl-lysine; OrAc: acetyl-ornithine; * the position of post-translational modifications are not indicated in Ref. [110].
